# The Chernobyl Tissue Bank — A Repository for Biomaterial and Data Used in Integrative and Systems Biology Modeling the Human Response to Radiation

**DOI:** 10.3390/genes3020278

**Published:** 2012-05-09

**Authors:** Geraldine Thomas, Kristian Unger, Marko Krznaric, Angela Galpine, Jackie Bethel, Christopher Tomlinson, Mark Woodbridge, Sarah Butcher

**Affiliations:** 1 Department of Surgery and Cancer, Imperial College London, Hammersmith Hospital, Du Cane Road, London W12 0HS, UK; E-Mails: k.unger@imperial.ac.uk (K.U.); m.krznaric@imperial.ac.uk (M.K.); a.galpine@imperial.ac.uk (A.G.); j.bethel@imperial.ac.uk (J.B.); 2 Bioinformatics Support Service, Imperial College London, Sir Ernest Chain Building, Division of Molecular Biosciences, South Kensington Campus, London SW7 2AZ, UK; E-Mails: chris.tomlinson@imperial.ac.uk (C.T.); m.woodbridge@imperial.ac.uk (M.W.); s.butcher@imperial.ac.uk (S.B.)

**Keywords:** Chernobyl, Tissue Bank, systems biology

## Abstract

The only unequivocal radiological effect of the Chernobyl accident on human health is the increase in thyroid cancer in those exposed in childhood or early adolescence. In response to the scientific interest in studying the molecular biology of thyroid cancer post Chernobyl, the Chernobyl Tissue Bank (CTB: www.chernobyltissuebank.com) was established in 1998. Thus far it is has collected biological samples from 3,861 individuals, and provided 27 research projects with 11,254 samples. The CTB was designed from its outset as a resource to promote the integration of research and clinical data to facilitate a systems biology approach to radiation related thyroid cancer. The project has therefore developed as a multidisciplinary collaboration between clinicians, dosimetrists, molecular biologists and bioinformaticians and serves as a paradigm for tissue banking in the omics era.

## 1. Introduction

Cancer is an extremely complex disease that involves the interaction of biological pathways on a number of levels. Activation of the individual pathways does not necessarily occur in an independent fashion through parallel linear routes, but operates through large and complex networks of interacting pathways. Interactions between pathways can occur at a number of different levels and can interact directly e.g., via phosphorylation events or indirectly e.g., via regulation of gene expression. 

In addition to understanding the interaction of pathways within a cell, it is clear that cancer cells do not exist within a vacuum. They respond to signals from outside of the individual cell, between different types of cell within a tissue (e.g., epithelial cells responding to signals generated from endothelial or stromal cells), and with stimuli external to the tissue e.g., hormones *etc.* Therefore cancer can be considered to be an extremely complicated system, one in which when one key node is blocked by use of an antineoplastic agent, for example, the system has the opportunity to re-route the signaling to overcome the block.

Most cancer researchers use rather reductionist approaches and focus their studies either on a particular gene of interest, or a particular pathway. The ability that we now have through the generation of “omics” data to provide data on multiple pathways simultaneously means that we need a paradigm shift in cancer research. The ability to generate several types of “omics” data from each individual cancer specimen will provide much more information about the system in general. This necessitates the ability to provide analytes of different types to be used in individual “omics” platforms to generate data on genome sequence, DNA copy number, epigenomic, transcriptomic, proteomic and metabolomic data. The data then needs to be integrated to identify the key genes and pathways driving an associated phenotype, such as drug response or clinical outcome.

Tissue banks may play a key role in this shift in our approach to the characterization of cancer by not only supplying human biosamples to researchers using complimentary technologies, but also providing a platform for the data on an individual patient to be collated and correlated with clinical information.

The Chernobyl Tissue Bank was established in 1998 and designed with a systems biology approach to radiation induced thyroid cancer in mind. This paper sets out the strategy for the development of the CTB and reviews the results of the project so far.

The Chernobyl accident happened on 26th April 1986, when an experiment went disastrously wrong. The resultant explosion and fire in the graphite core led to the release of more than 10^19^ Bequerels (Bq) of radioisotopes including 1.8 × 10^18^ Bq of 131-iodine, 2.5 × 10^18^ 133-iodine , and 1.1 × 10^18^ 132-Tellurium, which decays to 132-iodine [1]. It was the largest release of radioiodine into the environment and the radiation exposure of the population was quite different from that of the atomic bombs in Japan. In Japan, many people were killed by the blast from the bomb and those that survived received mainly external radiation. The most pronounced risk of thyroid cancer in those exposed to radiation from the atomic bomb was found in those exposed under the age of 10 years and the highest risk 15–29 years after exposure; an increased risk was still present 40 years after exposure [2]. In contrast, the routes of exposure after the Chernobyl accident were largely those of inhalation or ingestion of radionuclides. The thyroid is the only organ in the body to concentrate and bind iodine; exposure to the thyroid from 131-iodine is 1,000–2,000 times the average body dose [3]. 131-I has a short physical half-life (8.02 days), which results in quick elimination from the environment. Patients who were born more than 9 months after the accident were therefore not exposed to radioiodine either in utero or as young children. 

**Figure 1:**
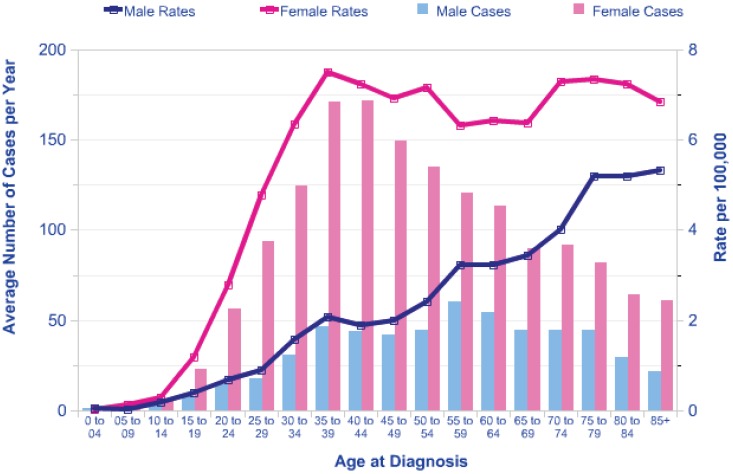
Thyroid cancer Average Number of New Cases per Year and Age-Specific Incidence Rates, UK, 2006–2008. Data available from http://info.cancerresearchuk.org/ cancerstats/types/thyroid/incidence/#age.

Many of the children in the exposed areas of Belarus, Ukraine and Russia received thyroid doses in excess of 1Gy [4]. The BEIR VII model of the risk of radiation induced thyroid cancer [5] based on studies of groups of children exposed to external sources of radiation [6] predicts a lifetime Excess Relative Risk (ERR) of around 10 per Gy for exposure in early childhood, falling to an ERR of about 2 per Gy at age 20 at exposure. There is a marked increase in background risk of thyroid cancer with attained age and the shape of this increase varies with gender. In females in the UK the incidence rises to a plateau of around 6 cases per 100,000 at an attained age of 40 years, and in males there is a steady rise in incidence with age (see Figure 1). This pattern is similar in other countries.

The current risk models suggest that there is no attenuation of the ERR with attained age. This suggests that there will be an inevitable relative increase in the number of cancers in the exposed population as the affected cohort ages. Although the increase in incidence is large, differentiated thyroid cancer diagnosed in the young has a good prognosis, and a recent review [7] has suggested that although there is a predicted recurrence rate of 30%, the death rate is predicted to be of the order of 1%. The data presented here only reflect cancer incidence, but there is evidence from the Ukraine-American cohort study that benign tumors of the thyroid are also increasing in incidence as a result of the radiation exposure [8] although at a lower rate than cancers. Whilst not life-threatening, these lesions require surgery to confirm absence of neoplasia and consequently have a significant health impact on the individuals concerned.

Whilst having serious consequences for the population, particularly those under 19 years old living in the immediate vicinity of the power station at the time of the accident, and those involved in the early phases of the clean-up of the accident and therefore exposed to high levels of radiation from the fallout, the Chernobyl accident provides a unique opportunity to collect samples of a human cancer, with a low natural incidence, for which both the etiology and the time of exposure to the etiological agent is known. Thyroid cancer is normally rare in children; of the order of 1 per million per year, although there is 50% variation in this figure across the globe [5].

## 2. Results and Discussion

### 2.1. The Chernobyl Tissue Bank—The Paradigm for a Cancer Resource Designed for Systems Biology

The CTB has been funded by four sponsors (the National Cancer Institute of the US, the European Commission (EC), the Sasakawa Memorial Health Foundation of Japan (SMHF) and the World Health Organization (WHO)) and is supported by two of the countries most affected by fallout from the reactor accident; Ukraine and the Russian Federation. 

In the early 1990s, a number of projects studying the effect of the Chernobyl accident were funded by the four sponsors listed above. The increase in thyroid cancers in children and adolescents in Belarus and Ukraine was confirmed in a number of publications [9,10]. By 1995 it was becoming apparent that several European research groups were unknowingly receiving material from the same patients for research, and that there were discrepancies in the pathological diagnoses being applied to the same tumor. Subsequently, a report to the EC confirmed that there had indeed been considerable overlap since 1995 among a number of EC-funded molecular biology projects [11]. It was then recognized that a cooperative tissue bank would reduce the duplication of research effort and provide better scientific data on the health effects of the Chernobyl accident. Following agreement on the various protocols, the Chernobyl Tissue Bank officially started collecting material on 1st October 1998. The full history and detail on the ethics and governance of the project have already been published [12].

The CTB study cohort comprises all patients with thyroid carcinomas and cellular follicular adenomas from the contaminated oblasts (the Russian and Ukrainian equivalent of a US county) of the Russian Federation (Bryansk, Kaluga, Tula and Oryol) and Ukraine (Kiev, Kiev city, Cherkasse, Chernigov, Rovno, Zhitomyr and Sumy) who were born on or after 26th April 1967 (*i.e.*, aged under 19 at the time of the Chernobyl accident) and operated on or after the 1st October 1998. In addition, a number of cases have been collected from other areas of Ukraine, relatively less contaminated by radioactive fallout. The collection currently comprises 3,861 cases of thyroid cancer and cellular follicular adenoma from patients who were under 19 at the time of the Chernobyl accident. Frozen material is available on 2,456 of these cases, and DNA and RNA has already been extracted from a quarter of these cases. Collection of blood samples began in late 1999 and samples of serum and whole blood have been collected from around 2,000 patients. One important feature of the project is that it also collects biosamples from patients resident in the areas of Ukraine and Russia exposed to radioactive fallout, but who were not exposed to radioiodine, as they were born more than 9 months after the accident. These cases form an age- and residency-matched cohort of patients who develop spontaneous thyroid neoplasia. This is the ideal cohort for comparison with those who were exposed to radioiodine in 1986. The current number of cases in this valuable cohort is 328 (206 with a diagnosis of cancer), with a further 182 (147 with cancer) coming from areas other than the exposed oblasts. This number is much lower than those exposed to radioiodine—the incidence of thyroid cancer being approximately the same as the background spontaneous rate from uncontaminated regions—of the order of 1 per million per year.

The current project consists of two banks of biological material and information comprising:

- Snap frozen and formalin fixed, paraffin embedded samples from tumor, normal tissue and, where possible, metastatic tissue from post operative specimens,
- nucleic acids extracted from these specimens,
- vials of serum from patients whose thyroid tissue is held in the bank,
- samples of whole blood,
- DNA extracted from blood
- Results from research projects supplied with samples from the CTB

### 2.2. Data Management within the CTB

**Figure 2:**
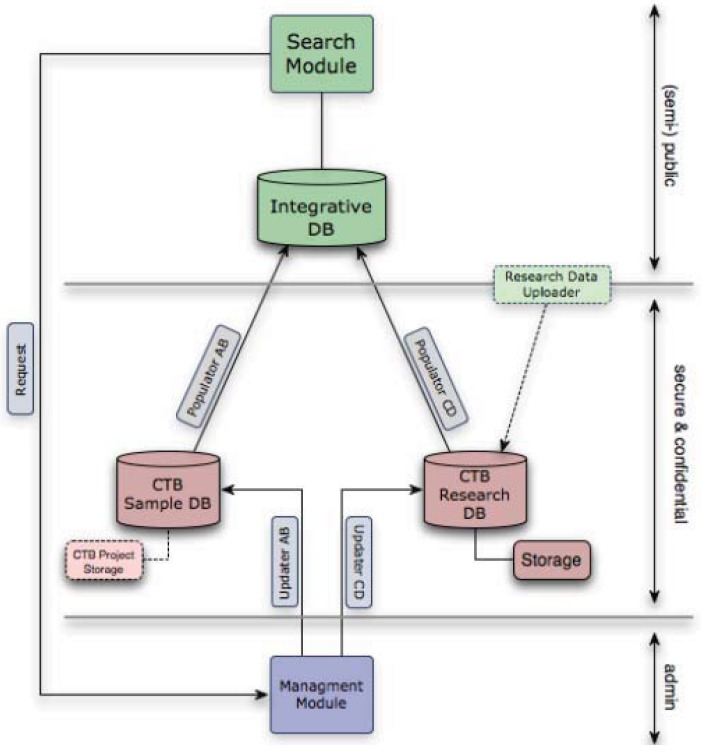
The Chernobyl Tissue Bank (CTB) data warehouse combines the Samples, Research and Integrative Databases, Management and Search Modules with corresponding front-ends/interfaces. The system was developed with assistance from the Bioinformatics Support Services (BSS) at Imperial College and continued close cooperation is essential for the smooth and secure integration of all the components of the overall system. Access to further samples and to information stored in the research database from the use of previous samples in research from the same patient is provided by the CTB portal.

The data management infrastructure for the CTB was designed to facilitate a systems biology approach to thyroid cancer. It comprises two separate databases, plus an integrated database that serves as a portal for researchers to access information on samples and data held and to apply for access to both data and samples (Figure 2).

One, centralized web-accessible database, held on secure servers at Imperial College London holds anonymised information on donors to the CTB and the biological samples donated by them. Regular, automated transfer of patient data back to secure servers in Ukraine and Russia ensures that each center has a local mirror copy. 

Detailed standard operating procedures for the collection and documentation of specimens and blood samples have been agreed upon with professional staff involved in the collection of material, and ethical standards agreed upon with the relevant authorities, conforming to the requirements of each country involved and those of the funding organisations. Each donor is identified by a unique alphanumeric code. Samples from each donor are identified by suffixes to this code enabling the specimens and any derivatives from them to be linked to the tissue block they were derived from and the individual donor. Tissue and blood samples, and extracted materials are recorded within tables in the CTB database. The database schema allows easy transfer of data between different database systems such as IBM DB2, Oracle, Microsoft SQL Server, MySQL and PostgreSQL. 

The samples database holds relevant information on the patient (date of birth, date of operation, sex, oblast of residence at the time of the accident and operation) together with pathological information and location coordinates for each sample of tissue, DNA or RNA extracted from tissue, and blood, serum and DNA extracted from blood, and information on the quality assurance of these derivatives is also recorded within the samples database. Dosimetry information, for each patient is held in the research database that also includes more detailed pathology information (e.g., subtype of tumor) and the research results fed back to the project by researchers using the samples.

Security and integrity of the data in the CTB Data Warehouse is of paramount importance. With regards to the Samples database and corresponding front-end, the access to data sets is appropriately restricted according to a country (e.g., Ukraine, Russia) and a role (e.g., pathologist, lab technician, administrator) to which a user belongs. Integration of the Samples database with the rest of the system and transfer of data between different elements of the overall CTB Warehouse are submitted to the same strict security requirements and are designed to minimize the risks of data loss or theft. Regular, time and place restricted updates of the Integrative database from the Samples and Research databases provide the up-to-date link between the research data and the key clinical-data elements required by a researcher.

### 2.3. The CTB Portal (https://cisbic.bioinformatics.ic.ac.uk/ctb/html_ctb_public/)

**Figure 3:**
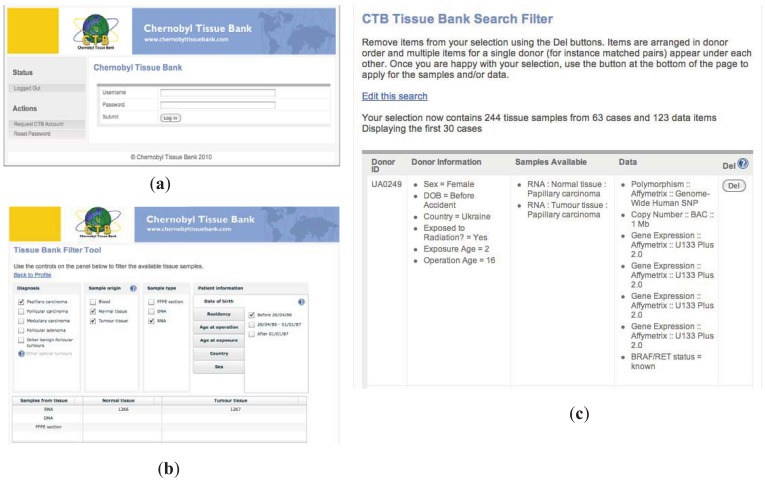
Representative screen shots of the CTB portal. (**a**). login entry page; (**b**). user searches samples by selecting criteria of interest. As search filters are selected, numbers of samples matching criteria are shown; (**c**). representative search results page showing available samples, search criteria and additional data available for sharing.

The CTB Portal provides on-line access to the resources of the CTB. Accessible either directly or from the CTB web site, the Portal gives access to a simple, but powerful, search facility which allows a researcher to search the database and check if samples are available that match the requirements for their study. Access is password protected to ensure security. Biomaterials are defined by: the type of thyroid tumor (from the consensus diagnosis of the Pathology Panel), the origin of the sample—blood, tumor tissue or normal tissue, the type of sample—FFPE section, extracted DNA or RNA and key patient information such as age at exposure, residency *etc.* The results of the search show the number of cases that match the criteria and the number of samples that are available. The system went live on the 25th anniversary of the Chernobyl accident. Representative screen shots of the Portal and the search filter are shown in Figure 3. 

The PI is guided through the on-line application process to request samples. Management tools embedded within the Portal, and accessible only by the secretariat, facilitate the processing of applications and tracking progress through the review and approvals process. Once the applicant has submitted their application on-line, the secretariat checks that the application is complete. The status of the application is altered as it progresses through each stage of the process and an E-Mail is automatically sent to the PI acknowledging each change of status: (submitted for review, more information required *etc.*). The software automatically compiles the various sections of the application into a pdf. The secretariat then forwards this to the External Review Panel for assessment of the scientific quality of the project requesting access. 

The integrative database produces a comprehensive list of all the cases identified that match the search criteria entered by the applicant. This listing is available only to the secretariat and is a significant step in the automation of the process of selecting appropriate samples for a project. The process can never be totally automated and expert oversight of the pathological information will always be required. However, the initial screening facilitates this procedure. The CTB Portal also provides access to the Data Warehouse both for PIs to upload data from their approved CTB projects and for other researchers to be able to see if data is available linked to the cases they have selected (see Figure 3).

### 2.4. Use of CTB Samples in Research

Biospecimens from the CTB have been provided to the major research groups involved in the studies of the consequences of the Chernobyl accident. Information on the projects receiving biosamples can be found at http://www.chernobyltissuebank.com/research.html. Twenty-seven projects have been approved for access to date; 11,254 samples have already been released to these projects. Scientific evaluation of each project is provided by the CTB’s External Review Panel (ERP) and the outcome of the review and, where appropriate, any feedback from the ERP is provided to the applicant.

This approach provides a basis that fosters international collaboration and reduces the chance of competition and even friction between groups in their requests for this material. Researchers who obtain material from the resource agree to provide the results of their investigation on a case-by-case basis to enable combined analysis to be carried out at a later date. The provision of extracted nucleic acid from thyroid tissue, rather than each researcher being provided with a small piece of tissue, maximises the amount of data that can, potentially, be obtained from a single operative specimen and enables multiple molecular biological studies to be carried out for each case. The median number of projects supplied by material from a single case at present is 4 with some being used in more than 9 projects. Details of the publications resulting from material supplied by the project are listed on the project website (www.chernobyltissuebank.com/papers.html). Data on Copy Number Alteration, mRNA expression, SNP and mutation/translocation of thyroid cancer key oncogenes RET and BRAF is already available for over a quarter of the cases, with miRNA array and methylation DNA array data being made available through the EU funded EpiRadBio project. 

#### 2.4.1. Pathological Studies

The pathology of all cases submitted to the CTB is reviewed by an International Pathology Panel. Their review of the cases has suggested a new classification for thyroid cancer [13] and has shown that the latency affects the morphological subtype and aggressivity of papillary cancers [14] with the frequency of the solid subtype falling from 24 to 6% over the first two decades after the accident, and a similar decline in the frequency of extrathyroid extension and nodal metastasis [15]. The Panel findings also report that the pathomorphology of the radiation-associated cancers is not different from an age- and residency-matched population, although the frequency of thyroid cancer in that population is markedly lower. It is highly unlikely that radiation dose therefore has any effect on the pathomorphology of the tumors induced by exposure to radiation. Interestingly, the pathomorphology of young-onset thyroid cancer in Ukraine (whether radiation induced or not) is different from that in Japan, suggesting that the pathomorphology may reflect differences in iodine content in the diet [16].

#### 2.4.2. Molecular Biological Studies

The molecular biology of post Chernobyl thyroid cancer has been reviewed recently [17]. Briefly, these studies suggest that the molecular biology of post-Chernobyl childhood thyroid cancer is broadly similar to that seen in age-matched series from non-irradiated populations. Post-Chernobyl papillary thyroid carcinomas, in common with non-radiation associated childhood papillary carcinomas, do not harbor RAS or p53 mutations [18,19] or show specific microsatellite instability [20]. However, three studies have now indicated that post Chernobyl thyroid cancers may show gains and losses of chromosomal material when DNA is analyzed on a global scale [21,22,23]. These earlier studies lack an appropriate age-matched control population. However, the most recent age-matched study using CTB samples [21] suggests that a specific amplification on chromosome 7q is associated with radiation exposure. Validation of this amplification is currently being carried out in a new project, using a further set of age matched cases and a series of cases with recorded dosimetry information. This project will examine both the association of the amplification with radiation exposure and seek to confirm that CLIP2, one of the genes coded for in this region of amplification and up-regulated at the mRNA level, is a key gene in radiation associated papillary thyroid cancer.

A number of studies have recently published transcriptomic analyses (reviewed by Maenhaut *et al.* [24], demonstrating different expression profiles between normal follicular thyroid epithelium or follicular tumors and papillary carcinomas [25,26,27,28,29]. However, these methods have not yet been shown to be able to differentiate between different types of papillary carcinomas and in one recent paper, it has been shown that the overall profile of post-Chernobyl papillary cancers is similar to papillary carcinomas from Belgium and France [30]. An updated analysis suggests that there are subtle differences between these two groups [31]. However, the two groups used in these studies were not age-matched and the data should therefore be interpreted with caution. Further studies are now underway using CTB samples to link transcriptomic studies with genomic changes in an age-matched population and in the series of tumors collected from patients recorded in the CTB that were also part of the Ukraine-American cohort. 

Studies are also underway to examine the effect of radiation exposure on the epigenetics (miRNA expression and methylated DNA) of thyroid cancer. Further studies are planned to extend the data available to include data from next generation sequencing platforms on selected cohorts within the CTB. The data from all of these studies will be available through the CTB portal to other researchers wishing to use samples from the same donors for their own research.

## 3. Conclusions

Understanding the major drivers in tumour growth will depend increasingly on being able to take a systems biology approach, rather than an individual gene or analytical platform approach. It is already evident from the literature that a change in copy number at the DNA level does not always result in an increase in RNA expression of all of the genes coded for by the amplified region, and that identifying critical nodes within networks of converging signalling pathways will be necessary to understand the functional biology of cancer [32]. Collating all of the research data generated from the samples donated by those affected by the Chernobyl accident is a challenge. By collating the data in a central resource, the CTB will facilitate not only projects that require access to samples alone, but also projects that wish to link their results with data already available. Tissue banks are likely to be key players in this type of cancer research in future, and should be designed from the outset to facilitate this aim.
